# Mapping the TAR vRNA Interaction with HIV-1 Integrase

**DOI:** 10.3390/v18060657

**Published:** 2026-06-09

**Authors:** Jian Sun, Rahul Yadav, Tolga Catmakas, Luke Fisher, Nicholas C. Fitzkee, Jacques J. Kessl

**Affiliations:** 1 Department of Chemistry and Biochemistry, University of Southern Mississippi, Hattiesburg, MS 39406, USAlfisher@rollins.edu (L.F.); 2 Department of Chemistry, Mississippi State University, Mississippi State, MS 39762, USA; rahul.yadav@uafs.edu (R.Y.); nfitzkee@chemistry.msstate.edu (N.C.F.)

**Keywords:** HIV, integrase, virus maturation, vRNA

## Abstract

A series of critical interactions within the viral core between viral RNA (vRNA) and HIV-1 Integrase (IN) has previously been reported. In these studies, contact points between vRNA and IN were identified using RNA-seq and MS-based protein foot-printing approaches. Several IN amino acids located in its C-terminal domain (CTD) were found to be essential for vRNA binding, and their alanine substitution severely impacted the correct morphogenesis of the mature viral core. Here, we have used the TAR element to extend these studies by performing a comprehensive mapping of the interaction by deploying RNA crosslinking and NMR methodologies. Together, these approaches were able to identify additional contact points between the TAR vRNA and IN. Our results reveal several new basic amino acids located in the IN CTD critical for the vRNA-IN interaction, viral replication and correct morphology of the mature viral core.

## 1. Introduction

The Integrase protein (IN) of Human Immunodeficiency Virus type 1 (HIV-1) inserts reverse-transcribed viral DNA (vDNA) into the host chromosome. This catalytic activity of IN, essential for the early stage of viral replication, has been developed as a therapeutic target, and several FDA-approved inhibitors (Raltegravir, Elvitegravir, Dolutegravir, Bictegravir and Cabotegravir) [1] are now commonly used to treat HIV-1-infected individuals. IN is composed of three structurally distinct domains: the N-terminal domain (NTD), the catalytic core domain (CCD) and the C-terminal domain (CTD) [2,3]. During integration, these three domains work in conjunction to form large and stable multimeric structures called intasomes, where vDNA ends are captured by IN [4,5,6,7,8]. The CCD subsequently inserts vDNA into the host chromatin through two sequential magnesium-dependent reactions, 3′-processing and strand transfer. Several mutational studies have revealed that various amino acid substitutions across the IN sequence can influence not only vDNA integration but also other viral replication steps [9,10]. Consequently, IN mutations have been grouped into two classes: substitutions that selectively impair integration have been assigned to class I mutations, and IN mutants that adversely affect multiple steps of virus replication have been grouped as class II [10]. Interestingly, some class II IN mutations can negatively impact correct virus particle maturation and infectivity during the late stage of replication. A hallmark of this phenotype is the mis-localization of the ribonucleoprotein complexes (RNPs) to an eccentric position trapped between the empty capsid (CA) core and the particle membrane in mature virions, whereas normal virions contain RNPs within the CA core. Characterizations of this viral phenotype have been performed either through electron microscopy [11,12] or sucrose gradient fractionation [13]. Intriguingly, allosteric IN inhibitors (ALLINIs [12,13,14,15,16,17], which are also known as LEDGINs [18], NCINIs [19], or INLAIs [20]) selectively bind at the IN CCD dimer interface, induce aberrant IN multimerization and consecutively yield non-infectious particles with eccentrically positioned RNPs similar to those seen with several class II IN mutants [11]. Together, these observations strongly suggest that IN not only directs vDNA integration but also contributes to the maturation step during viral egress. Using an array of in vitro and ex vivo approaches, we and others have previously reported a strong and essential interaction between viral RNA (vRNA) and HIV-1 IN within the virion [12,21]. Additionally, these studies [12] have pointed to the significant contribution of the IN CTD. Here, we have performed a comprehensive mapping of the IN-vRNA interaction using both vRNA crosslinking and NMR methodologies. These approaches are able to identify new functionally essential contact points between the vRNA and IN. Taken together, our results reveal that several basic amino acids located in the IN CTD are critical for the TAR vRNA-IN interaction and the correct morphology of the mature viral core.

## 2. Materials and Methods

*DNA Constructs and Recombinant Proteins.* Alanine substitutions were introduced using PCR-site directed mutagenesis (Agilent, Santa Clara, CA, USA) and verified by DNA sequencing. For biochemical studies recombinant proteins 6×His-tagged full length and CTD domain of WT (residues 220-270) and mutant INs were expressed in *E. coli* and purified by column chromatography as previously described [22,23]. Briefly, cell pellets were resuspended in 1000 mM NaCl (for full length) or 500 mM NaCl (for CTD), 20 mM Imidazole, 7.5 mM CHAPS, 2 mM BME, and 25 mM HEPES, pH 7.4; sonicated; and centrifuged at 26,000 *g* for 30 min at 4C to remove insoluble material. The lysate was then purified with a Ni-NTA column (GE Healthcare, Chicago, IL, USA) using a linear gradient of 50 to 500 mM imidazole (in the same corresponding buffers). Fractions containing target proteins were pooled, diluted with 300 mM NaCl, 7.5 mM CHAPS, 2 mM BME, and 25 mM HEPES, pH 7.4, and purified with a Heparin column (GE Healthcare, Chicago, IL, USA) using a linear gradient of 300 to 1000 mM NaCl (in the same buffer). Fractions containing the target protein were pooled. All proteins were checked for purity by SDS-PAGE, quantified by UV absorbance, and stored in 10% glycerol at −80 °C until used. When not needed, the His tag was removed with PreScission protease digestion before the Heparin column step. ^13^C^15^N-CTD protein was expressed in minimal media M9 supplemented with 1 g/L ^15^NH4Cl and 2.5g/L ^13^C_6_-glucose.

*IN-RNA Crosslinking for MS/MS.* A 57 mer TAR vRNA substrate with a 4-thiouridine (4sU) modification at base 30 was synthesized and obtained from Horizon Discovery (UK). The IN-TAR complex was allowed to form by incubating 4µM IN and TAR vRNA in a 1:1 ratio for 30 min at RT in 100 mM NaCl, 1 mM DTT, 0.8 mM CHAPS, 3% glycerol, and 25mM HEPES, pH 7.4. Crosslinking was performed on ice by subjecting the samples to 30 min of 365 nm UV irradiation at 600 mJ/cm^2^ (Analytik Jena UVP Crosslinker, Jena, Germany). Crosslinking efficiency was monitored by either PAG-SDS or Western blot using monoclonal IN antibodies (NIH AIDS Reagent Program, Bethesda, MD, USA). Crosslinked samples were then incubated with RNase I (Invitrogen, Carlsbad, CA, USA) for 30 min at 37 °C, acetone-precipitated and Trypsin-digested overnight at 37 °C to yield peptides with RNA fragments of ~1–2 bases covalently linked.

*Tandem mass spectrometry (MS/MS) analysis.* Trypsin-digested samples were analyzed at the Louisiana State University Mass Spectrometry facility using electrospray ionization (ESI) MS/MS using a Thermo Scientific Q-Exactive mass spectrometer system (Thermo Fisher Scientific, Waltham, MA, USA). Briefly, approximately 1 µg of the sample was injected and eluted using an acetonitrile/0.1M acetic acid gradient with a flow rate of 0.3 µL/min for 2 hours. The nanospray ion source was utilized at a voltage of 1.9 kV. RAW data files were processed with the Open search algorithm [23] to identify the crosslinked amino acid residue. As the traditional search engines for modifications require the adduct to be stable during the tandem mass spectrometry (MS/MS)—and the fragmentation profile of the crosslinked peptides makes up complex data, where the adduct mass of the crosslinked species can change during analysis [24]—the Open search algorithm within the MSFragger search engine [25] was utilized. This method provided a comprehensive analysis, where every mass shift observed in the MS/MS portion of the data was analyzed. Once the adduct was identified, isolated MS/MS data peaks were exported via the MZmine 3 software v3.9.0 [26] and confirmed using the ProteinProspector MS-Product tool (http://prospector.ucsf.edu) (accessed on 1 January 2026).

*AlphaScreen-Based RNA Binding Assays.* Direct binding of WT or mutant INs to RNA molecules was performed as previously described [12]. Briefly, 50 nM 6xHis-tagged INs were incubated with increasing concentrations of synthetic biotinylated RNAs (Integrated DNA Technologies, Coralville, IA, USA) at 4C for 1 h in AlphaScreen buffer containing 100 mM NaCl, 1 mM MgCl_2_, 1 mM DTT, 1 mg/mL BSA, and 25 mM Tris, pH 7.4. The final concentration of the nickel and streptavidin AlphaScreen beads (PerkinElmer, Shelton, CT, USA) in each well was 20 mg/mL. The AlphaScreen signal was recorded on an EnSpire Plate Reader (PerkinElmer, Shelton, CT, USA) with an AlphaScreen module using the instrument presets. Data were fitted to a Hill equation using the Origin software (OriginLab, Inc., Northampton, MA, USA).

*NMR and Chemical Shift Perturbation Mapping of CTD-TAR Complex.* All HSQC (heteronuclear single quantum coherence) NMR experiments were performed on uniformly ^15^N-labeled CTD protein samples prepared in buffer containing 100 mM NaCl, 8 mM CHAPS, 10 mM DTT, 3% glycerol, and 25 mM HEPES, pH 7.4, on a 600 MHz Bruker Avance III NMR system equipped with a CP-QCI cryoprobe. Two-dimensional ^1^H–^15^N HSQC-TROSY spectra were recorded at 25 °C using the standard HSQC Bruker pulse sequences optimized for transverse relaxation-optimized spectroscopy (TROSY) [27]. Spectra were acquired with 1024 complex points in the direct (^1^H) dimension and 512 increments in the indirect (^15^N) dimension. Spectral widths were set to 14.026 ppm (8417.51 Hz) for ^1^H and 34.000 ppm (2067.825 Hz) for ^15^N. The acquisition times were 121.7 ms in the direct dimension and 123.8 ms in the indirect dimension. A 1.5 s recycle delay was used between scans, and 32 scans were collected per increment. Water suppression was achieved using sensitivity-enhanced gradient HSQC with flip-back water suppression. The amide resonance in the 2D ^1^H–^15^N HSQC spectrum of the CTD was initially assigned based on previously reported NMR data [22]. As we observed some slight variations in the published assignment (most likely due to buffer mismatch), amide backbone resonances were confirmed by collecting and analyzing 3D triple-resonance NMR spectra (HNCA, HNCO, CBCANH, and CBCA(CO)NH) collected on a uniformly ^15^N,^13^C-labeled CTD sample. Backbone resonance assignment of INT-CTD resulted in the identification of 46 out of 49 non-proline backbone amide resonances. Because assignments were largely consistent with those already published [22], only the backbone amide chemical shifts are reported here (Supplementary Materials). Chemical shift perturbations were collected in the presence of an increasing amount of synthetic and unlabeled TAR RNA. After each addition, changes in chemical shifts of the protein resonances were monitored in TROSY ^1^H–^15^N HSQC spectra. A total of four CTD/RNA ratios (150 µM CTD) were examined: 1:0.1, 1:0.2, 1:0.3 and 1:0.4. Spectral data were processed using NMRPipe [28] and analyzed with NMRFAM-SPARKY [29].

*Viruses and Cells.* HIV-1_NL4-3_ molecular clone was used to generate replication-competent HIV-1 virions. The mutant viruses were made by introducing the respective substitutions into the IN coding sequence by site-directed mutagenesis (Agilent). HEK293T and HeLa TZM-bl cells were grown in Dulbecco’s modified Eagle medium (Invitrogen), supplemented with 10% fetal bovine serum (Invitrogen), penicillin (100 I.U/mL) and streptomycin (100 mg/mL) (GIBCO) at 37C and 5% CO_2_.

*Transfection and Virion Preparations.* Transfections in HEK293T cells were performed with X-tremeGENE HP (Roche, Basel, Switzerland) and the desired plasmid DNA (1:3 for DNA:reagent ratio). Cell culture supernatants containing virions were harvested 48 h post-transfection and filtered through a 0.45 µm filter, and virions were concentrated by ultracentrifugation with a 25% sucrose cushion at 28,000 rpm for 2 h at 4C in an SW41 rotor (Beckman, Brea, CA, USA). Isolated virions were assayed by HIV-1 Gag p24 ELISA (ZeptoMetrix, Buffalo, NY, USA) to determine viral production. HeLa TZM-bl target cells were then infected with quantities of virions equivalent to 4 ng of HIV-1 p24 using 12-well plates. Infected cells were cultured for 48 h, and cell extracts were prepared using a reporter lysis buffer (Promega). Levels of infectivity were monitored in TZM-bl reporter cells by assaying luciferase activity with a Luciferase Assay Kit (Promega, Fitchburg, WI, USA) and normalized by total protein concentration, as previously reported [13].

*HIV-1 Viral Core Analyses Using Sucrose Density Gradient Fractionation.* For sucrose density gradient fractionation [12,13], cell-free virions from transfected HEK293T cells were concentrated by ultracentrifugation over a 25% sucrose cushion, pelleted virions were lysed with 0.5% Triton X-100 and were centrifuged through a 30–70% linear sucrose density gradient. Fractions were collected starting from the top of the gradient and subjected to immunodetection with a commercial HIV-1 p24 monoclonal antibody (SinoBiological, Beijing, China) to monitor the distribution of HIV-1 capsid.

*Molecular Modeling*. The computer-based binding model was generated using a Biovia Discovery Studio 2024 software package (Dassault Systèmes Biovia Inc., San Diego, CA, USA) on an Alienware Area 51 R2 workstation. The previously published 3D structures of the CTD and the TAR vRNA stem loop (PDB ID: 1IHV [22] and 1LVJ [30], respectively, both obtained through NMR experiments) were used as starting coordinates. A rigid-body docking approach using the ZDOCKER module was used to generate a binding pose consistent with the experimental binding data obtained from both the crosslinking and the NMR-based approaches. A minimization stage was used to prepare the complex using a CHARMM-based forcefield (2000 steps at steepest descent followed by 3000 steps at conjugate gradient). The structure was then subjected to a Standard Dynamic Cascade (Heating, Equilibration and Production) followed by a second minimization. The obtained 3D structure was visualized using PyMOL 3.1 (Schrodinger, New York City, NY, USA).

## 3. Results

**Binding site crosslinking of vRNA TAR construct into full-length IN**

**Figure 1:**
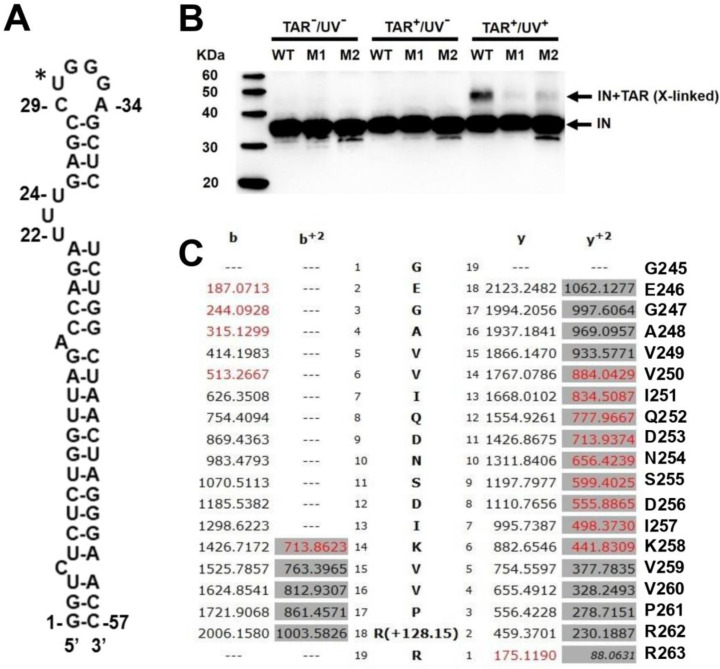
IN-TAR crosslinking. (**A**) TAR sequence with star label at position 30. (**B**) Western blot of full-length IN proteins after UV-crosslinking experiment using modified 4sU(30)-TAR vRNA construct. The R269A/K273A (M1) and K264A/K266A (M2) IN mutants were used as negative controls. (**C**) Mass spectrometry-based sequencing of the GEGAVVIQDNSDIKVVPRR peptide showing the R262 crosslinked position.

WT recombinant full-length IN and a synthetic RNA construct containing a single photoreactive nucleobase were used to pinpoint the orientation and position of the vRNA during viral maturation. While several regions of the viral genome have been shown by CLIP-seq to interact with IN ex vivo [12], the vRNA_(1–57)_-trans-activation response element (TAR) of the viral genome was selected as the interacting ligand (i) because of its stable and simple structure and (ii) because it displays high in vitro affinity toward IN [12]. For those reasons, TAR has been used in previous studies as an in vitro surrogate of IN-vRNA-binding activities [12,31,32]. In this experiment, we designed and synthesized a TAR substrate that substitutes 4-thiouridine (4sU) at position 30 (Figure 1A—star-labeled base). This position on vRNA has been reproducibly identified as a primary crosslinking site within the TAR region during CLIP-seq experiments through the T-to-C base changes induced by the reverse transcription step [12]. Previously published K264A/K266A and R269A/K273A IN mutant constructs [12], which have been shown to be TAR-binding defective, were used here as negative controls (Figure 1B). Upon binding, 4sU-subtituted TAR covalent crosslinking with full-length recombinant IN was initiated by controlled UV irradiation [24]. The formation of the IN-TAR crosslinked adduct was monitored by either PAGE-SDS or Western blot (Figure 1B) and showed a mass shift of +17kDa, corresponding to the covalent addition of the RNA construct. This adduct was then subjected to RNase A/T1 treatment and trypsin proteolysis to yield peptides with RNA fragments of ~1–2 bases covalently linked. Analysis and sequencing of these IN peptides using tandem mass spectrometry (MS/MS) approaches (see Section 2) detected the formation of IN R262 residue crosslinked to the 4-thiouridine at position 30 with an added mass of 128.15 Da (Figure 1C), corresponding to 4-Thiouracil. Therefore, this sequencing data strongly suggests that within the IN-TAR complex, nucleotide 30 of TAR is positioned in close proximity to the IN R262 residue as the RNA substrate interacts with several surface-exposed and positively charged IN residues.

**Mapping TAR binding to IN CTD using protein NMR**

**Figure 2:**
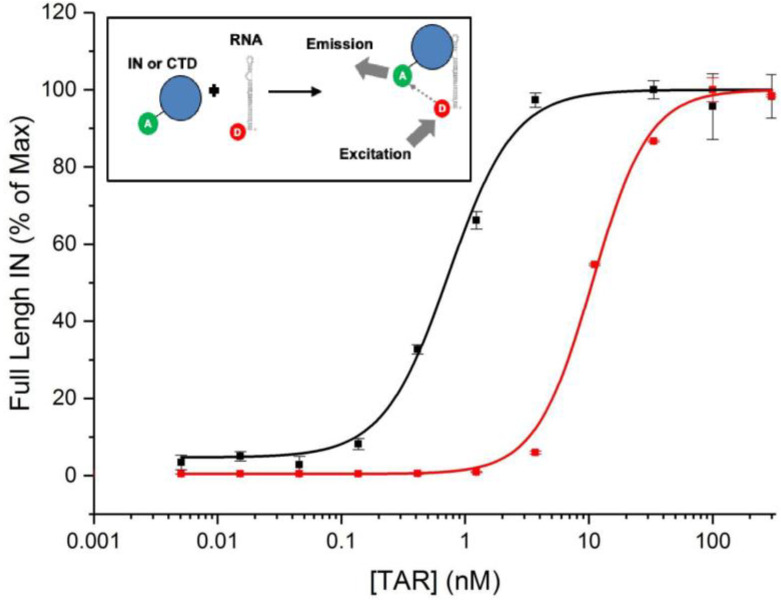
AlphaScreen-based binding affinity measurements of vRNA TAR fragment against full-length IN (black) or IN-CTD (red). Assay schematic illustrated in top-left window. Error bars represent the standard deviation from three independent replicates.

**Figure 3:**
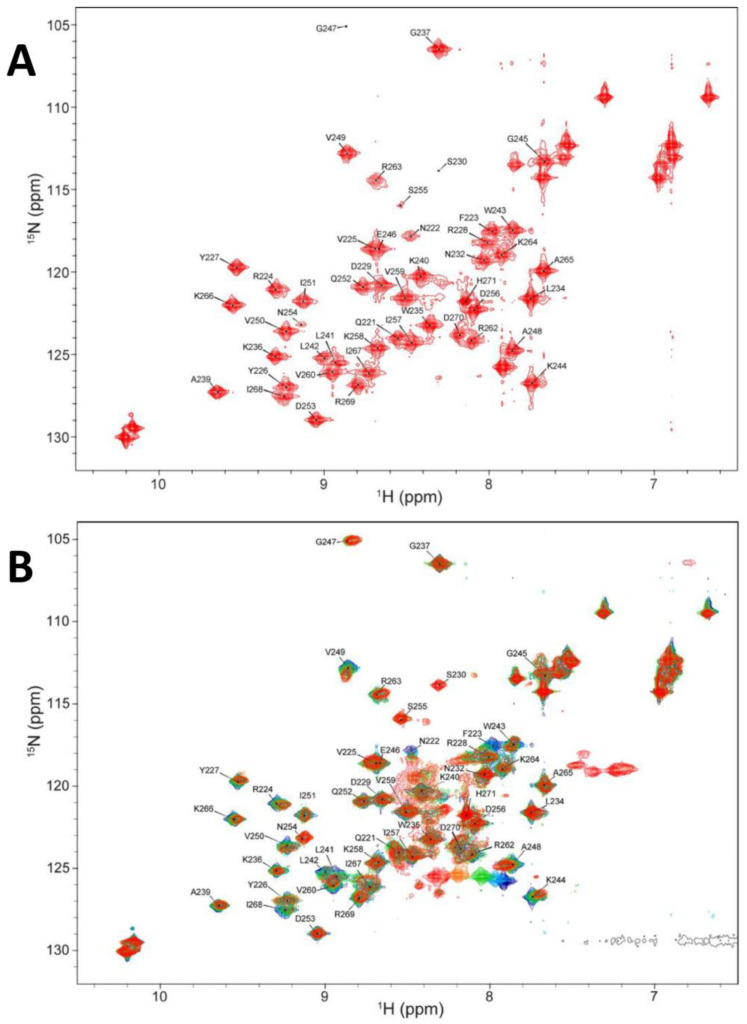
2D Protein NMR of the CTD-TAR complex. (**A**) Signal assignments confirmed by ^15^N/^13^C double-labeling experiment with CTD alone. (**B**) Spectrum of ^15^N labeled CTD IN with chemical shift perturbations induced by TAR titration with four CTD/RNA ratios: 1:0.1 (cyan), 1:0.2 (green), 1:0.3 (orange) and 1:0.4 (red).

As the IN-TAR crosslinking study above and previously published observations [12,32] suggest, contact points between vRNA and IN point to a major role of the IN CTD. To measure in isolation the specific contribution of this particular IN domain to this interaction, we have used a modified AlphaScreen binding assay to compare side-by-side the binding affinity of the full-length IN and the CTD toward TAR (Figure 2). While this data confirms a significant binding between CTD and TAR, a lower affinity toward the RNA construct was observed with the IN domain compared to the full-length protein. This 10-fold difference suggests either a folding variation of the CTD when expressed in isolation or the presence of several additional contact points outside the CTD. The binding properties between CTD and TAR were nevertheless compatible with the requirements for NMR detection. As NMR structures of CTD [22] have been previously reported by others, we have used published NMR amide resonance data (Figure 3A) to assign the obtained signals. In order to map the CTD residues interacting with TAR, we have monitored the chemical shift perturbations (CSPs) upon the formation of the CTD-TAR complex. For this, we have used a purified ^15^N-labeled CTD protein by expressing this construct in minimal media supplemented with ^15^N-ammonium chloride as the sole nitrogen source. To measure these CSPs, a synthetic unlabeled TAR RNA construct was titrated into the labeled CTD protein while simultaneously acquiring 2D ^1^H/^15^N HSQC (Heteronuclear Single Quantum Coherence) spectra (Figure 3B). As several backbone amide resonances shifted upon RNA additions, we were able to pinpoint several surface-exposed CTD residues (Figure 3B and Table 1) potentially contributing to the interaction with the TAR fragment. NMR resonance signals during the formation of a protein–ligand complex are known to be affected by the rate of exchange between free and bound forms relative to the frequency difference between these states [33,34]. When the rate is higher (“fast exchange”), a progressive change in peak position is observed across the titration, while when the rate is lower (“slow exchange”), separate free and bound resonances are observed with population-dependent intensities. Thus, analyses of exchange rate signals during titration (also referred to as dynamic NMR) [35] can be used to reveal binding kinetics on the CTD-TAR complex formation. As high-affinity complexes usually show “slow exchange” and low-affinity complexes show “fast exchange” [36], different residues exhibiting spectral behavior consistent with fast and slow exchange on the NMR timescale were observed during titration (Table 1). The residues Q221, R228 and R263 displayed slow exchange kinetics, while F223, K244, R262, K264 and K266 showed fast rates (Table 1).

**In vitro validation of HIV-1 IN residues interacting with TAR**

**Figure 4:**
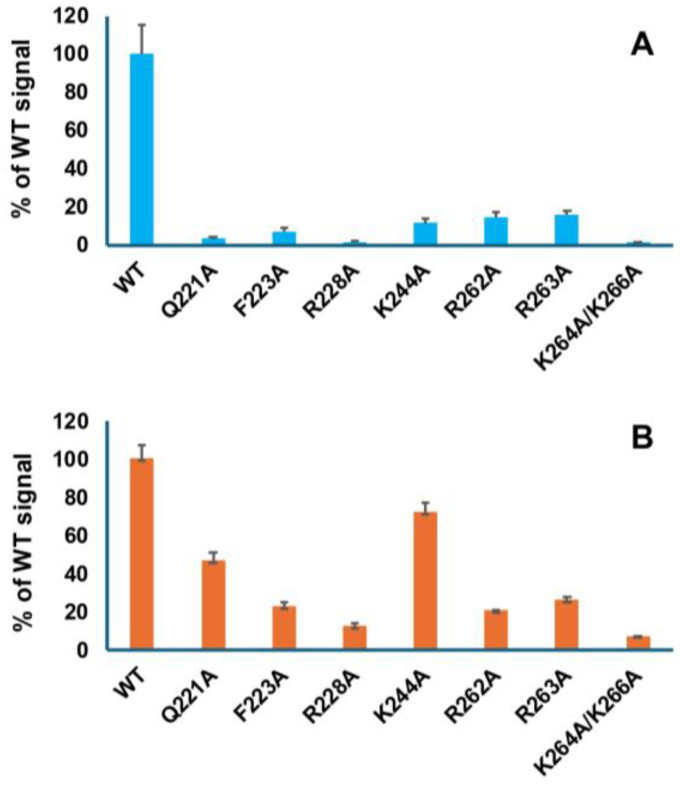
Effect of Alanine substitution on vRNA TAR *in vitro* binding to full-length IN or IN-CTD using AlphaScreen-based assay. (**A**) CTD. (**B**) Full-length protein. Values were normalized against WT IN (100%). Error bars represent the standard deviation from three independent replicates.

To quantify and validate the contribution of the residues identified above (from both the 4sU crosslinking and the protein NMR experiments) to TAR binding, we have created recombinant mutated CTD and full-length IN proteins with single Alanine substitutions at these specific locations. Each mutated construct was evaluated for its binding affinity toward TAR using the AlphaScreen in vitro assay described above (Figure 2) and compared to the WT proteins. For this, WT or mutated IN proteins (full-length or CTD constructs) were incubated with biotinylated TAR before the addition of AlphaScreen beads. As the K264A/K266A double-mutant IN protein has been previously shown to be defective in TAR binding [12], we have used this protein as a control. To show the contribution of these two residues, we have also introduced the K264A/K266A double mutation into the CTD construct. These in vitro measurements revealed that all tested mutations impacted TAR binding (Figure 4A,B). Notably, we observed that the impact of Alanine substitution at each tested position was more severe in the CTD construct (Figure 4A) than in the context of the full-length IN proteins (Figure 4B), strongly suggesting the contribution of additional contact points with the vRNA construct outside the CTD, as measured earlier (Figure 2).

**IN mutations impaired for vRNA binding yield non-infectious virions with eccentric core morphology**

**Figure 5:**
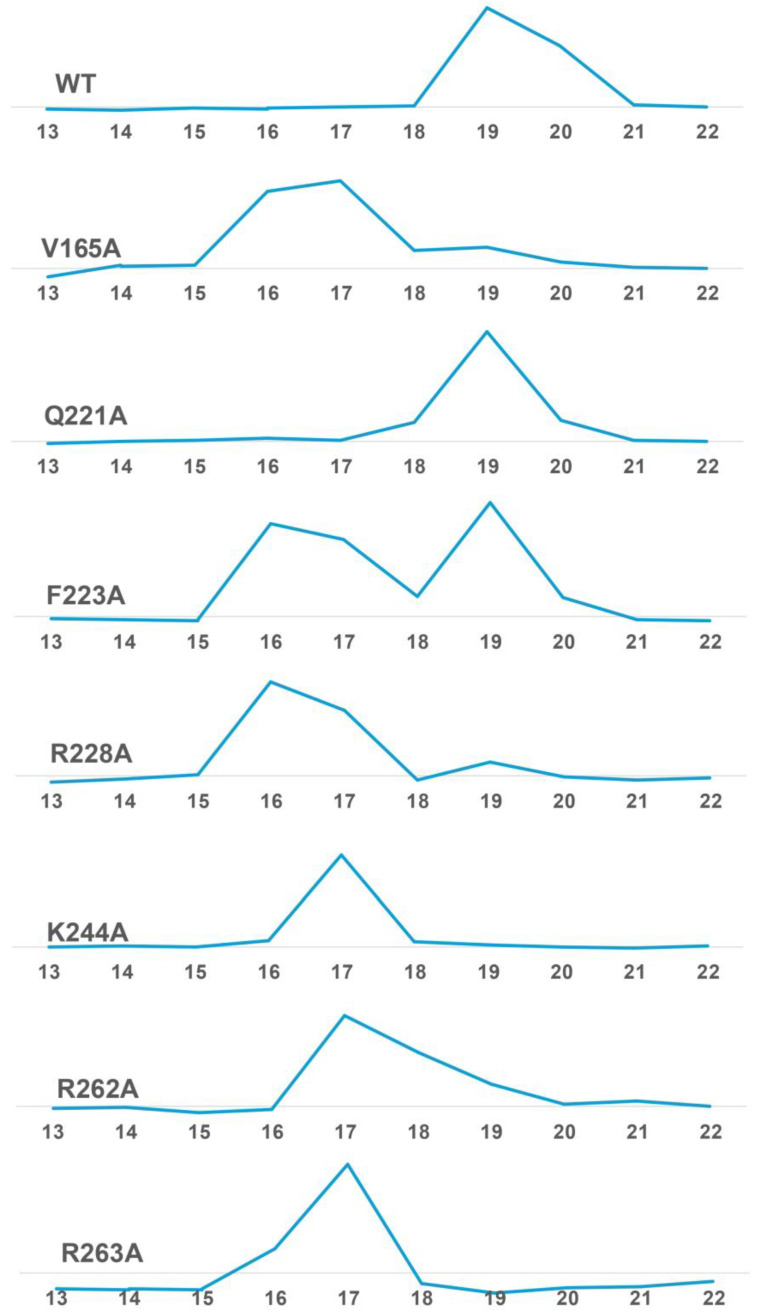
Viral core density profiles of IN WT and mutant virions observed by sucrose fractionation of detergent-lysed virions. The relative capsid (p24) distribution in the bottom fractions is represented. Horizontal axis indicates the fraction number.

**Figure 6:**
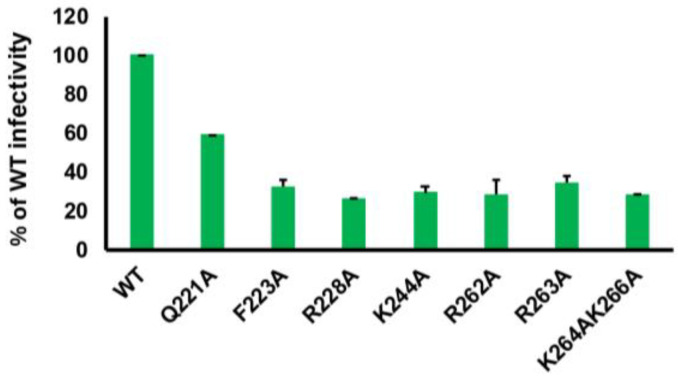
IN mutant virion infectivity measured in target cells. Values were normalized against WT (100%). Error bars represent the standard deviation from three independent replicates.

To further validate the biological significance of the identified CTD residues, we performed Alanine substitutions at the positions described above in the replication-competent HIV-1_NL4-3_ construct to examine their impact on capsid core morphology and viral replication. Previous studies, including ours and others, have shown that IN mutations affecting vRNA binding influence proper virus particle maturation, leading to the production of non-infectious particles with eccentrically positioned RNPs. This mislocalization of the RNP to a position between the low-density empty capsid core and the particle membrane in mature virions has previously been observed using electron microscopy [9,11,21,32]. Additionally, the buoyant sucrose gradient technique, which measures the relative density of the viral core in mature virions, has also been validated as an effective approach to characterize this mislocalization of the RNP [12,13,21,37]. To confirm the effect of these IN mutations on virus particle maturation, we subjected cell-free and detergent-lysed progeny virions to a linear 20 to 70% (wt/vol) sucrose gradient fractionation. The CA core density was then detected by measuring the HIV-1 CA (p24) content of each sucrose fraction. We used the previously described class II V165A mutation as a control, as it has consistently been shown to display the eccentric core phenotype [11]. The R228A [32] and the double-mutant R262A/R263A [21] have also shown an eccentric core phenotype through electron microscopy. This experiment revealed that specific CTD mutations resulted in a reduction in the CA signal in high-density fractions and a simultaneous increase in low-density fractions compared to the WT construct (Figure 5). This observation is consistent with the formation of empty cores due to the mislocalization of the RNPs, characteristic of the IN mutant class II phenotype [9]. Among the mutations tested, the F223A exhibited a bimodal distribution of p24 along the gradient (Figure 5), indicating the presence of both eccentric and correct core densities, suggesting a weak or indirect effect on viral morphogenesis. Interestingly, the construct carrying the Q221A mutation was the only one displaying a WT profile, suggesting a marginal impact on viral maturation. To further observe the impact of these IN mutations on viral replication, the infectivity of the virions isolated from transfected cells was measured in target cells and compared to the WT construct (Figure 6). Except for Q221A, which retained around 60% of WT infectivity, all the considered mutations had a decrease below 30%. Together, these experiments (Figure 5 and Figure 6) confirmed that single Alanine substitutions at the F223, R228, K244 and R263 positions in a replication-competent HIV-1_NL4-3_ construct significantly impact capsid core morphology and viral replication.

## 4. Discussion

Given its involvement observed at multiple stages of viral replication, the HIV-1 IN C-terminal domain (CTD) has been described as the virus’s “Swiss army knife” [38]. With its SH3-like beta-barrel fold structure, this sub-domain appears to perform a wide variety of functions. In early studies, this domain, characterized by the presence of several basic residues, showed substantial non-specific DNA-binding properties [39,40,41]. Recent intasome structures have pointed to the side chains of IN residues R228, K244, R262, R263, K264, K266 and R269 as components of a basic surface patch capable of interacting with vDNA [41]. A docking study supported by biochemical analysis suggests that INI1, a subunit of the cellular SWI/SNF chromatin remodeling complex, is incorporated into viral particles and also interacts with CTD [32]. Early studies have shown that various mutations within INs (class-II IN mutants) [9,10,42] could trigger the formation of a particular viral morphological defect in which the RNP condensates outside the capsid core (also described as the eccentric core phenotype). Notably, several of these IN mutants had their mutations located in the CTD [42].

These early observations strongly suggested that IN not only directs vDNA integration but also plays a functional role during viral maturation. More recently, IN has been observed to interact directly with vRNA during viral egress [12]. The interaction occurs at specific and reproducible sites on the viral genome, mostly on structured elements such as TAR. Preventing this interaction with vRNA by mutating IN causes the morphological defects described above.

In the present study, we have used a two-pronged in vitro-based strategy to probe and map the IN-vRNA molecular interface. We first used full-length recombinant IN and a high-affinity synthetic TAR 57-nucleotide construct that displays a 4sU modification at position 30 [12] to specifically crosslink and identify the IN residue located in the immediate vicinity of this nucleotide. After RNase treatment and trypsin proteolysis of the full-length protein, mass spectrometry-based sequencing reproducibly identified IN R262 residue as likely positioned adjacent to nucleotide 30 of TAR (Figure 6, magenta position). Notably, no other crosslinked IN residues were detected. As the IN-TAR crosslinking study above and previously published observations [12,32,42] suggest, contact points between vRNA and IN point to a major contribution from the IN CTD.

Because the NMR structure of CTD [22] has been previously reported, we have used this approach to identify additional RNA-interacting CTD residues by monitoring chemical shift perturbations (CSPs) upon the formation of the CTD-TAR complex. Using this method, we were able to pinpoint eight surface-exposed CTD residues (Q221, F223, R228, K244, R262, R263, K264 and K266) potentially contributing to the interaction with the TAR fragment. Several of those IN residues (R228, K244 and K266) have been previously observed exhibiting class II phenotypes when mutated into Alanine [42]. Interestingly, as discussed above, some of these IN residues (R228, K244, R262, R263, K264 and K266) have also been described as components of a basic surface patch contributing to the CTD interaction with vDNA during integration [38]. The contribution to the TAR binding by each position identified in this study (Q221, F223, R228, K244, R262, R263, K264, and K266) was measured in vitro using recombinant proteins (Figure 4) and ex vivo in HIV-1_NL4-3_ constructs to examine their impact on capsid core morphology and viral replication. While Alanine replacement in the CTD construct severely impacted TAR binding at all positions (Figure 4A), the effect was mitigated for Q221A and K244A when full-length proteins were tested (Figure 4B). Additional ex vivo studies confirmed that the infectivity of virions exhibiting the Q221A mutation was marginally impacted, with no observed defect in the viral core morphology. On the other hand, virions with the K244A IN mutation showed poor infectivity and capsid core density defect, confirming a possible direct contribution to vRNA binding. Interestingly, while other mutated virions (R228A, K244A, R262A and R263A) exhibited complete shifts toward low viral core densities, the F223A mutant was mixed, suggesting a weaker contribution.

While our study was in the late writing stage, a new approach exploring the possible morphology of the IN-RNA complex was published [43]. In this work, cryogenic electron microscopy (cryo-EM) was used to model the interactions between IN, TAR vRNA and the capsid core. Because of the insurmountable challenges caused by the spontaneous aggregation properties of HIV-1 IN at the concentrations needed with this approach, the authors substituted the HIV-1 protein with the cryo-EM-compatible SIVtal IN (55% identity with HIV-1 IN). Observation using cryo-EM of concentrated SIVtal IN protein vitrified in the presence of HIV RNA^TAR^ revealed the formation of elongated polymers assembled from protein octamers formed around multiple extended duplexes of RNA molecules instead of distinct hairpin-like TAR structures. While the authors acknowledged that the RNA duplex arrangements may have been caused by the specific experimental conditions required for cryo-EM and were unlikely to occur during maturation with the full viral genome, several CTD contact points observed with this approach (R228, K244, R262, R263, and K264) confirmed our above finding.

**Figure 7:**
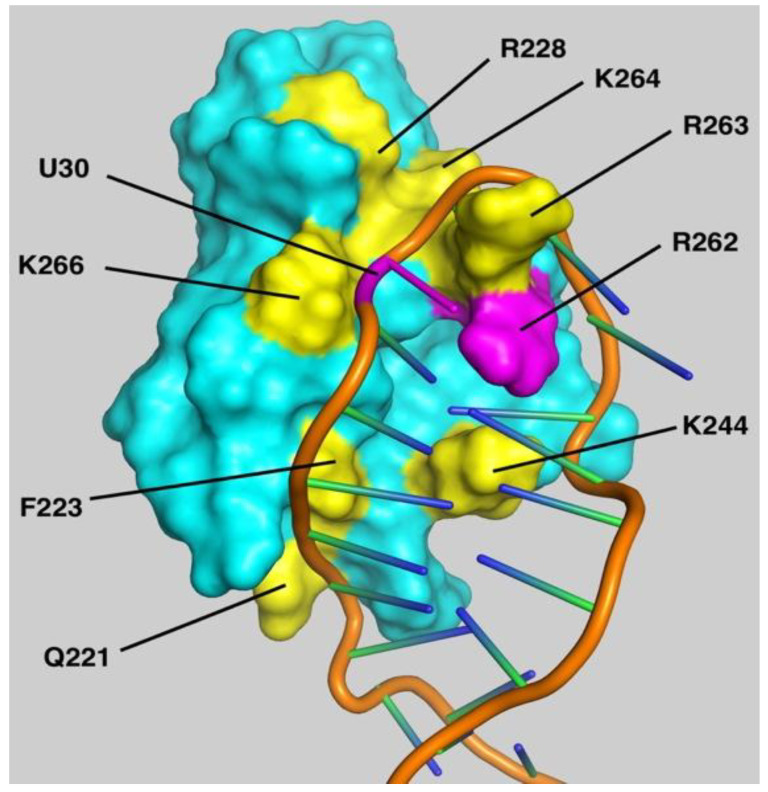
3D binding model of the CTD-TAR complex. CTD residues detected with NMR-CSP approach are colored in yellow. Crosslinked TAR nucleotide #30 and CTD R262 residue are colored magenta.

Using the data collected in this work, we have generated an energy-minimized molecular model of the CTD-TAR complex (Figure 7) that locates the crosslinked nucleotide U30 near R262 (Figure 7, magenta residue) and predicts the relative positions of the RNA fragments according to NMR shifts (Figure 7, yellow positions). Interestingly, the proposed model shows the TAR hairpin loop wrapping tightly around a positively charged post structure composed of R262 and R263. Alanine replacement at those positions either individually (this work) or in combination [21,42] severely impacts RNA binding, infectivity and virion morphology.

Of note, both Q221 and K244 are predicted (Figure 7) to interact with the ascending and descending stems of the TAR element, which could explain why NMR and the in vitro measurements with the truncated CTD protein may artificially amplify the binding contribution of these two residues compared to the full-length protein. More contact points are expected to occur outside the CTD construct used in this work. Most notable is the role of K34 within the IN NTD, which has been shown to contribute to vRNA binding [21]. By providing a new experiment-based model of the CTD-TAR complex (Figure 7), our study offers a molecular basis for previously observed class II mutation phenotypes, such as R228A [21], double-mutant R262A/R263A [21], and K264A/K266A [12].

## 5. Conclusions

We have performed a comprehensive mapping of the IN-TAR interaction in a solution state using two independent approaches: vRNA crosslinking and NMR-CSP. Eight surface-exposed CTD residues (Q221, F223, R228, K244, R262, R263, K264 and K266) potentially contributing to the interaction with the TAR fragment were detected by NMR-CSP. The contribution to TAR binding of these residues was measured both in vitro and ex vivo using single-Alanine substitutions. By crosslinking TAR to IN, we were also able to pinpoint the position of the stem-loop structure of TAR relative to the CTD by locating the nucleotide 30 close to the residue R262. Using the data collected in this work, we have generated a new working model that maps the binding interface of the CTD-TAR complex.

## Figures and Tables

**Table 1:** NMR exchange rates of identified CTD residues.

CTD Residue	Exchange Rate
Q221	Slow
F223	Fast
R228	Slow
K244	Fast
R262	Fast
R263	Slow
K264	Fast
K266	Fast

## Data Availability

All required data are available as texts and figures in the main text and the Supplementary Materials.

## Supplementary Materials

The following supporting information can be downloaded at https://www.mdpi.com/article/10.3390/v18060657/s1, Table S1: Backbone ^1^H, ^15^N resonance assignments for CTD.

## Abbreviations

The following abbreviations are used in this manuscript:

HIV-1	Human Immunodeficiency Virus type 1
IN	Integrase
vRNA	Viral RNA
TAR	Trans-activation response element
CTD	C-terminal domain
4sU	4-Thiouridine
CA	Capsid
RNP	Ribonucleoprotein
