# Supplementary material for: Mapping the TAR vRNA Interaction with HIV-1 Integrase
Source: Viruses. 2026 Jun 9;18(6):657. doi: 10.3390/v18060657 (PMC13307726; doi:10.3390/v18060657)
Supplement: Supplementary file 1 [file viruses-18-00657-s001.zip › viruses-4330118-supplementary.pdf]

Table S1. Backbone ^{1}H, ^{15}N resonance assignments for CTD.

	Amino acid	^{1}H (ppm)	^{15}N (ppm)
1	M219	NA	NA
2	I220	NA	NA
3	Q221	8.552	124.03
4	N222	8.477	117.842
5	F223	7.98	117.554
6	R224	9.294	121.061
7	V225	8.689	118.599
8	Y226	9.23	126.99
9	Y227	9.529	119.737
10	R228	8.008	118.222
11	D229	8.654	120.807
12	S230	8.307	113.861
13	R231	NA	NA
14	N232	8.031	119.3
15	P233	NA	NA
16	L234	7.748	121.606
17	W235	8.359	123.232
18	K236	9.295	125.14
19	G237	8.305	106.484
20	P238	NA	NA
21	A239	9.644	127.281
22	K240	8.414	120.27
23	L241	8.933	125.514
24	L242	8.998	125.223
25	W243	7.857	117.468
26	K244	7.74	126.748
27	G245	7.664	113.299
28	E246	8.668	118.604
29	G247	8.866	105.091
30	A248	7.862	124.764
31	V249	8.86	112.803
32	V250	9.23	123.595
33	I251	9.126	121.772
34	Q252	8.765	120.906
35	D253	9.05	128.985
36	N254	9.138	123.192
37	S255	8.537	115.976
38	D256	8.092	122.282
39	I257	8.471	124.29
40	K258	8.676	124.626
41	V259	8.509	121.585
42	V260	8.95	126.068
43	P261	NA	NA
44	R262	8.107	124.164
45	R263	8.686	114.439
46	K264	7.924	118.96
47	A265	7.67	119.923
48	K266	9.554	122.012
49	I267	8.73	126.145
50	I268	9.241	127.542
51	R269	8.794	126.842
52	D270	8.177	123.82
